# Hypoxia Inducible Factor-1*α* Regulates the Migration of Bone Marrow Mesenchymal Stem Cells via Integrin *α*_4_

**DOI:** 10.1155/2016/7932185

**Published:** 2016-01-04

**Authors:** Jong Ho Choi, Yun Bin Lee, Jieun Jung, Seong Gyu Hwang, IL-Hoan Oh, Gi Jin Kim

**Affiliations:** ^1^Department of Biomedical Science, CHA University, Seongnam-si 13488, Republic of Korea; ^2^Department of Internal Medicine, CHA Bundang Medical Center, CHA University, Seongnam-si 13496, Republic of Korea; ^3^Department of Nanobiomedical Science, Dankook University, Cheonan-si 31116, Republic of Korea; ^4^Catholic High Performance Cell Therapy Center, The Catholic University of Korea, Seoul 06591, Republic of Korea

## Abstract

Although hypoxic environments have been known to regulate the migratory ability of bone marrow-derived mesenchymal stem cells (BM-MSCs), which is a critical factor for maximizing the therapeutic effect, the underlying mechanisms remain unclear. Therefore, we aimed to confirm the effect of hypoxia-inducible factor-1*α* (HIF-1*α*) on the migration of BM-MSCs and to analyze the interaction between HIF-1*α* and integrin-mediated signals. Hypoxia-activated HIF-1*α* significantly increased BM-MSC migration. The expression of integrin *α*_4_ was decreased in BM-MSCs by increased HIF-1*α* under hypoxia, whereas the expression of Rho-associated kinase 1 (ROCK1) and Rac1/2/3 was increased. After downregulation of HIF-1*α* by YC-1, which is an inhibitor of HIF-1*α*, BM-MSC migration was decreased via upregulation of integrin *α*_4_ and downregulation of ROCK1 and Rac1/2/3. Knockdown of integrin *α*_4_ by integrin *α*_4_ siRNA (siITGA4) treatment increased BM-MSC migration by upregulation of ROCK1, Rac1/2/3, and matrix metalloproteinase-2 regardless of oxygen tension. Moreover, siITGA4 treatment increased HIF-1*α* expression and augmented the translocation of HIF-1*α* into the nucleus under hypoxia. Taken together, the alternative expression of HIF-1*α* induced by microenvironment factors, such as hypoxia and integrin *α*_4_, may regulate the migration of BM-MSCs. These findings may provide insights to the underlying mechanisms of BM-MSC migration for successful stem cell-based therapy.

## 1. Introduction

Mesenchymal stem cells (MSCs) are adult multipotent stem cells capable of differentiation into cells originating from any of the three germ layers, that is, the endoderm, mesoderm, and ectoderm [1]. Bone marrow is an abundant source of MSCs, and bone marrow-derived mesenchymal stem cells (BM-MSCs) have been extensively studied and determined to have the ability to differentiate into osteogenic, chondrogenic, and adipogenic lineages with the capability to migrate toward injured tissues and sites of inflammation [2–5]. Recently, MSCs have been applied in an increasing number of clinical trials for the treatment of various diseases based on their proven efficacies in preclinical and clinical studies [6, 7]. To maximize the therapeutic efficacy of stem cell therapy using BM-MSCs, several requisite characteristics should be established: (1) a high survival rate and a high proliferative potential of transplanted cells; (2) effective homing properties; and (3) sufficient interactions between grafted MSCs and environmental factors at sites where MSCs have migrated [8]. Among these factors, effective homing, which is the capability of MSCs to migrate into target sites, is the most important and challenging characteristic to achieve [9].

Generally, the homing mechanisms of MSCs have been shown to be similar to those of inflammatory cells [10]. Proinflammatory factors released from damaged tissues induce the production of chemokines, cytokines, and the expression of adhesion molecules [11, 12]. Kim et al. reported that pretreatment with tumor necrosis factor-*α*, a cytokine involved in acute inflammation, enhanced the adhesiveness and migration of MSCs through the overexpression of bone morphogenetic protein-2 [13]. Furthermore, stromal cell-derived factor 1*α* (SDF-1*α*), which is also known as C-X-C motif chemokine 12, has been shown to play a crucial role in cell-cell adhesion, adhesion to extracellular matrix (ECM), and cell migration [14, 15].

Hypoxia-inducible factor-1*α* (HIF-1*α*) is a key mediator of the adaptive cellular response to hypoxia and is upregulated under hypoxic conditions, modulating the expression of numerous genes that affect cellular survival and metabolism [16]. Recently, it was determined that MSC migration was enhanced under hypoxic conditions through the increased expression of chemokine receptors, such as CX3C chemokine receptor 1, C-X-C chemokine receptor type 4 (CXCR4), and SDF-1*α* [17]. Therefore, hypoxia preconditioning was tried and revealed to enhance the survival and engraftment of MSCs in previous* in vitro* and* in vivo* studies [18–20].

The homing capabilities of MSCs are also affected by the dynamic expression of integrins, which are heterodimeric transmembrane proteins composed of *α* and *β* subunits that regulate cell-cell adhesion, cell differentiation, and migration [21]. Saller et al. suggested that low oxygen concentration augments the stemness and migration of MSCs and alters integrin expression [22]. However, the underlying mechanisms through which altered integrin expression under hypoxia affects the migration of MSCs have not been fully elucidated.

The Rho GTPase family consists of three members: Rac1, RhoA, and Cdc42 [23]. Rho GTPases have been determined to be associated with a variety of cellular processes, especially in the regulation of cytoskeletal dynamics and cell migration [24]. The overexpression of RhoA in noninvasive cells gives rise to the increased invasiveness, whereas the inhibition of RhoA suppresses cellular invasiveness by modulating downstream signals, such as Rho-associated kinase (ROCK) and myosin light chain [25]. However, it is still unclear whether the Rho family of GTPases influences the migration and homing of MSCs.

Therefore, we aimed to analyze the effect of increased expression of HIF-1*α* on the migration of BM-MSCs and to assess the alterations of integrins and Rho GTPases in BM-MSCs under hypoxia. Moreover, we evaluated the correlation between HIF-1*α* and integrin *α*_4_ in BM-MSCs.

## 2. Materials and Methods

### 2.1. Cell Culture

Human BM-MSCs were purchased from Lonza, Ltd. (Wakersville, MD, USA) and maintained in *α*-MEM (Gibco-BRL, Grand Island, NY, USA) supplemented with 10% fetal bovine serum (FBS), 2 mM L-glutamine (Gibco-BRL), 100 U/mL penicillin, and 100 *μ*g/mL streptomycin (Gibco-BRL) at 37°C in a 5% CO_2_ incubator which contains 20% O_2_ (normoxic condition). To analyze the effect of hypoxia, the cells were cultured in a hypoxic chamber (C-chamber, BioSpherix, Ltd., Lacona, NY, USA) by lowering the oxygen concentration to 1% for 24 hours. Additionally, BM-MSCs were treated with YC-1 (20 *μ*M) (AG Scientific Inc., San Diego, CA, USA) under normoxic or hypoxic conditions for 24 hours to downregulate HIF-1*α*.

### 2.2. Downregulation of Integrin *α*_4_ in BM-MSCs Using Short Interfering RNA (siRNA)

BM-MSCs were treated with integrin *α*_4_ siRNA (siITGA4) (Santa Cruz Biotechnology, Santa Cruz, CA, USA) or mock to inhibit integrin *α*_4_ expression according to the manufacturer's protocol under normoxic or hypoxic conditions. Briefly, 4 *μ*L of Lipofectamine 2000 (Invitrogen, Carlsbad, CA, USA) containing 100 pM of siITGA4 was added to the cells. The cells were cultured for 24 hours under normoxic or hypoxic conditions.

### 2.3. Quantitative Real-Time Polymerase Chain Reaction

Total RNA was extracted from BM-MSCs using TRIzol reagent (Invitrogen). Reverse transcription was performed with 500 ng of total RNA and Superscript III reverse transcriptase (Invitrogen). Real-time polymerase chain reaction (PCR) was performed with SYBR EX taq (Roche, Mannheim, Germany). The cDNA was amplified by PCR using the following thermal conditions: 5 minutes at 95°C, 40 cycles at 95°C for 30 seconds, 60°C for 15 minutes, 70°C for 15 minutes, and 72°C for 7 minutes. 18S rRNA (Bioneer, Seoul, Korea) was used as internal control for normalization. The sequences of the primers used were as follows: HIF-1*α* (NM_001243084.1) forward 5′-CTT CGA TCA GTT GTC ACC AT-3′, HIF-1*α* reverse 5′-TCC ATA CGG TCT TTT GTC AC-3′, integrin *α*_4_ (NM_000885.4) forward 5′-AGA GAG ACA ATC AGT GGT TGG-3′, integrin *α*_4_ reverse 5′ TCA GTT CTG TTC GTA AAT CAG G-3′, 18S rRNA (NR_003286.2) forward 5′-GTA ACC CGT TGA ACC CCA TT-3′, and 18S rRNA reverse 5′-CCA TCC AAT CGG TAG TAG CG-3′. All reactions were conducted in triplicate.

### 2.4. Immunofluorescence

To investigate the expression of HIF-1*α* and integrin *α*_4_, BM-MSCs (5 × 10^4^ cells/well) seeded onto coverslips were pretreated with 20 *μ*M of YC-1, which is a specific inhibitor of HIF-1*α*, and cultured under normoxic or hypoxic conditions for 24 hours. The cultured cells were washed with cold phosphate-buffered saline (PBS) and incubated with serum-free protein blocking buffer (Dako, Glostrup, Denmark) at 37°C for 1 hour followed by incubation with mouse anti-HIF-1*α* antibody (1 : 100, Santa Cruz Biotechnology) or mouse anti-integrin *α*_4_ (1 : 100, Novus Biologicals, Littleton, CO, USA) overnight at 4°C. After washing with PBS, the cells were incubated with a secondary antibody conjugated to Alexa 488 or Alexa 588 (1 : 500, Invitrogen) for 1 hour at room temperature. Then, the cells were stained with 4′,6-diamidino-2-phenylindole (DAPI) for nuclear counterstaining and observed under a fluorescence microscope at a magnification of 400x (Axioskop2, Carl Zeiss Microimaging, Germany).

### 2.5. Western Blot Analysis

BM-MSCs were lysed on ice with RIPA buffer cocktailed protease inhibitor (Roche) and phosphates inhibitor (Sigma-Aldrich, St. Louis, MO, USA). The protein lysates were separated via 8%–15% sodium dodecyl sulfate polyacrylamide gel electrophoresis (SDS-PAGE) and subsequently transferred to polyvinylidene difluoride membranes. The membranes were incubated overnight at 4°C with the appropriate primary antibodies. The following primary antibodies were used: rabbit anti-HIF-1*α* (1 : 1,000, BD Biosciences, San Jose, CA, USA), rabbit anti-integrin *α*_4_ (1 : 1,000, ProSci-Inc., Poway, CA, USA), rabbit anti-integrin *α*_5_ (1 : 1,000, BD Biosciences), mouse anti-integrin *β*_7_ (1 : 1,000, R&D Systems, Minneapolis, MN, USA), rabbit anti-RhoA (1 : 1,000, Cell Signaling Technology, Danvers, MA, USA), rabbit anti-ROCK1 (1 : 1,000, Cell Signaling Technology), rabbit anti-Rac1/2/3 (1 : 2,000, Cell Signaling Technology), rabbit antiphosphorylated Rac1/cdc42 (1 : 1,000, Cell Signaling Technology), and rabbit antiphosphorylated focal adhesion kinase (FAK) (1 : 500, Cell Signaling Technology). Then, the membranes were reacted with a peroxidase-conjugated secondary antibody (anti-rabbit IgG [1 : 25,000, Bio-Rad Laboratories, Hercules, CA, USA] or anti-mouse IgG [1 : 10,000, Bio-Rad Laboratories]) for 1 hour at room temperature. The bands were detected using an enhanced-chemiluminescence reagent (Amersham Biosciences, Piscataway, NJ, USA).

### 2.6. Invasion Assay

The invasiveness of BM-MSCs was analyzed using 24-well filtered inserts with membranes (8 *μ*m pore size; Thermo Fisher Scientific, Rockford, IL, USA) on 24-well plates. In the inserts, 3 × 10^4^ cells were seeded and treated with serum-free Opti-MEM (Gibco-BRL) containing siITGA4 or YC-1. Culture medium containing FBS was added in the lower well and incubated for 24 hours under normoxic or hypoxic conditions. After incubation, the cells in the upper wells were completely removed with a cotton swab. The invading cells that had attached to the bottom side of the filter were fixed with methanol for 20 minutes and stained with Mayer's hematoxylin (Sigma-Aldrich) for 20 minutes. The cell invasion ability was determined by counting the number of stained cells attached to the other side of the filter in seven randomly selected fields on the membranes at a magnification of 100x. Cell invasion under different treatments was normalized to controls and expressed as the mean invasion (% invasion ± SEM).

### 2.7. Gelatin Zymography

To analyze the activities of matrix metalloproteinase- (MMP-) 2 and MMP-9, BM-MSCs were treated with YC-1 or siITGA4 and cultured under hypoxia. Then, the conditioned medium was analyzed by zymography. The conditioned medium was separated by 12% SDS-PAGE supplemented with 1 mg/mL of gelatin (Bio-Rad Laboratories). The separated proteins were incubated for 30 minutes using a renaturation buffer (Bio-Rad Laboratories), rinsed, and incubated in a development buffer (Bio-Rad Laboratories) at 37°C for 24 hours. The gels were stained with Coomassie Brilliant Blue R-250 solution for 2 hours at room temperature and then destained with a buffer comprised of 10% acetic acid, 30% methanol, and 60% deionized water until the zymogen bands were visualized. The activities of MMP-2 and MMP-9 were analyzed by the density of unstained bands. All experiments were performed in triplicate.

### 2.8. Statistical Analysis

Student's* t*-tests were performed for group-wise comparisons and a* P* value less than 0.05 was considered statistically significant. All experiments were performed in triplicate.

## 3. Results 

### 3.1. HIF-1*α* Induced by Hypoxia Enhances the Migration of BM-MSCs through Activation of MMP-2

**Figure 1:**
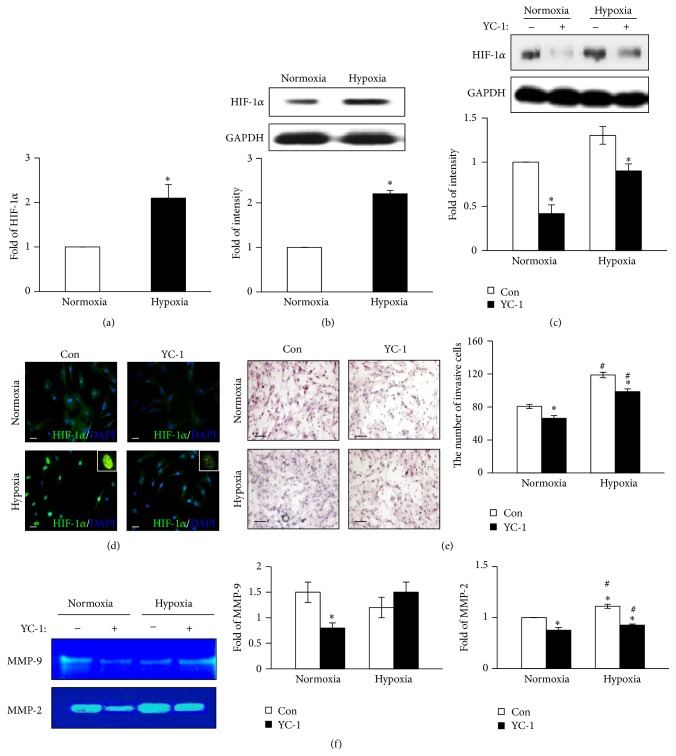
HIF-1*α* induction by exposure to hypoxia stimulates BM-MSC migration. (a) Real-time PCR analysis showing the transcription levels of HIF-1*α* in BM-MSCs under normoxic or hypoxic conditions. 18S rRNA was used as the loading control. (b) Protein expression levels of HIF-1*α* in BM-MSCs under normoxic or hypoxic conditions were analyzed by Western blotting. GAPDH was used as the loading control. (c) Protein expression levels of HIF-1*α* in BM-MSCs under normoxic or hypoxic conditions after pretreatment with YC-1, an inhibitor of HIF-1*α*, were analyzed by Western blotting. (d) Immunofluorescence staining showing localization of HIF-1*α* in BM-MSCs after YC-1 pretreatment under normoxic or hypoxic conditions. Blue: DAPI; green: HIF-1*α*. Scale bar = 80 *μ*m (400x original magnification). (e) Invasiveness of BM-MSCs after pretreatment with YC-1 determined by invasion assay (left). Quantification of the cells invaded through the inserts (right). (f) Enzymatic activities of MMP-9 and MMP-2 in BM-MSCs under the indicated conditions analyzed by zymography (left). Quantification of enzymatic activities of MMP-9 (middle) and MMP-2 (right). ^*∗*^ *P* < 0.05 (compared with YC-1 nontreated group) and ^#^ *P* < 0.05 (compared with normoxic group). DAPI: 4′,6-diamidino-2-phenylindole; HIF-1*α*: hypoxia-inducible factor-1*α*; MMP: matrix metalloproteinase.

To confirm the effect of hypoxia on the migration of BM-MSCs, we analyzed the expression of HIF-1*α*. When the cells were cultured under hypoxia, the transcription and expression of HIF-1*α* were significantly higher than those of cells cultured under normoxia (*P* < 0.05; Figures 1(a) and 1(b)). After the pretreatment with YC-1, an inhibitor of HIF-1*α*, the expression of HIF-1*α* significantly decreased under both normoxia and hypoxia (Figure 1(c)). The localization of HIF-1*α* into the nucleus, which indicates activation of HIF-1*α*, was more pronounced when the BM-MSCs were cultured under hypoxia. However, the translocation of HIF-1*α* in BM-MSCs was decreased under both normoxia and hypoxia when the cells were pretreated with YC-1 (Figure 1(d)). While the migration of BM-MSCs was significantly augmented under hypoxic conditions compared with normoxic conditions, the migration was suppressed by YC-1 pretreatment under both normoxic and hypoxic conditions (*P* < 0.05; Figure 1(e)). In a previous study, the increased activities of MMP-2 and MMP-9 were determined to contribute to BM-MSCs [26]; we therefore verified the changes in the activities of MMP-2 and MMP-9 by zymography. As shown in Figure 1(f), the activity of MMP-2 significantly increased under hypoxia compared with normoxia (*P* < 0.05), whereas the activity of MMP-9 did not significantly change. Collectively, these results suggest that the migration of BM-MSCs was directly regulated by HIF-1*α* through MMP-2 activation under hypoxic conditions.

### 3.2. Expression of Integrins and Rho GTPases in BM-MSCs under Hypoxia

**Figure 2:**
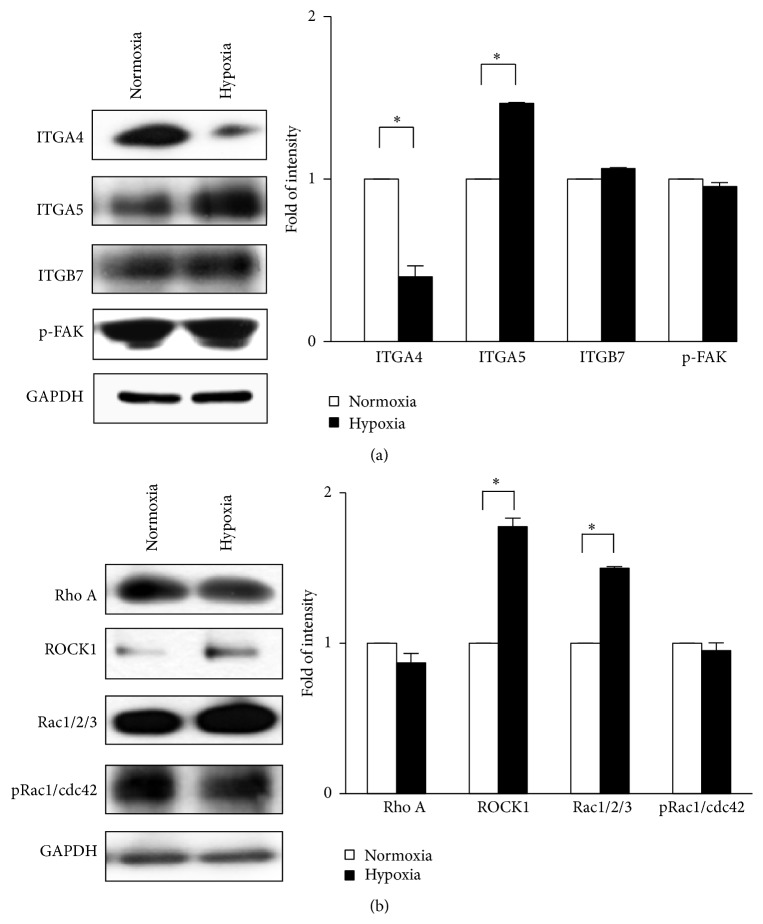
Expression of signaling molecules associated with cellular invasiveness including integrins and Rho GTPases under hypoxia. (a) Protein expression levels of integrins and phosphorylated FAK under normoxic or hypoxic conditions. (b) The expression levels of RhoA, ROCK1, Rac1/2/3, and phosphorylated Rac1/cdc42. GAPDH was used as the loading control. ^*∗*^ *P* < 0.05 (compared with normoxic group). ITGA4: integrin *α*_4_; ITGA5: integrin *α*_5_; ITGB7: integrin *β*_7_; p-FAK: phosphorylated focal adhesion kinase; pRac1/cdc42: phosphorylated Rac1/cdc42.

Generally, the dynamic expression of molecules involved in cell adhesion and cytoskeletal remodeling, such as integrins and Rho family proteins, through environmental signals is required for cellular migration. Therefore, we explored the effect of hypoxia on the migration ability of BM-MSCs through the alternative expression of adhesion molecules, including integrins, phosphorylated FAK, and Rho GTPases. Interestingly, the expression of integrin *α*_4_ was significantly lower when BM-MSCs were exposed to hypoxia, whereas the expression of integrin *α*_5_ was significantly higher under hypoxia (*P* < 0.05; Figure 2(a)). However, the expression of integrin *β*_7_ and phosphorylated FAK was not significantly different between cells cultured under normoxia and hypoxia (Figure 2(a)). Next, we analyzed the expression of Rho proteins. Although the expression of RhoA and phosphorylated Rac1/cdc42 was not changed by hypoxia, the expression of Rac1/2/3 and ROCK1, which is a downstream signal of integrins and Rho GTPase family, was augmented by hypoxia (*P* < 0.05; Figure 2(b)). These findings indicate that the alternative expression of integrin *α*_4_ and ROCK1 in BM-MSCs is induced by hypoxia. Moreover, integrin *α*_4_ could be negatively correlated with migration of BM-MSCs.

### 3.3. Inhibition of HIF-1*α* by YC-1 Increases the Expression of Integrin *α*_4_ in BM-MSCs regardless of Oxygen Concentration

**Figure 3:**
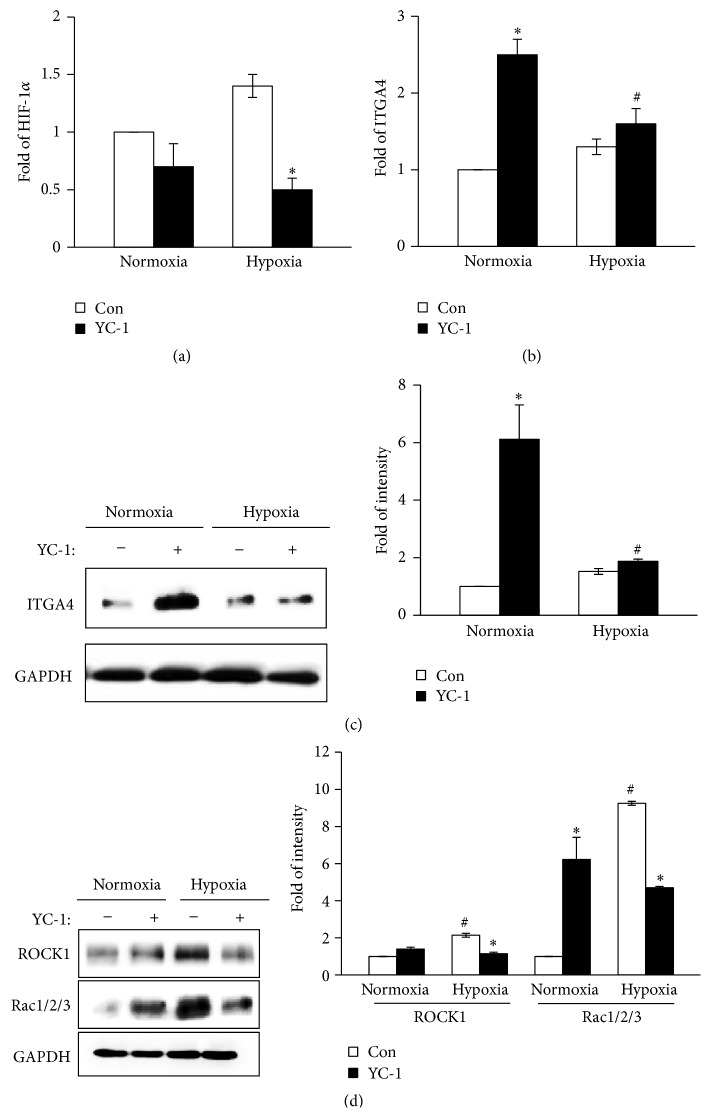
Alteration of integrin *α*_4_-mediated signaling pathway in BM-MSCs under hypoxia. The mRNA expression levels of HIF-1*α* (a) and integrin *α*_4_ (b) in BM-MSCs were determined by real-time PCR. 18S rRNA was used as the loading control. Protein expression levels of integrin *α*_4_ (c) and ROCK1 and Rac1/2/3 (d) in BM-MSCs were assessed by Western blotting. GAPDH was used as the loading control. ^*∗*^ *P* < 0.05 (compared with YC-1 nontreated group) and ^#^ *P* < 0.05 (compared with normoxic group). HIF-1*α*: hypoxia-inducible factor-1*α*; ITGA4: integrin *α*_4_; ROCK1: Rho-associated kinase 1.

To evaluate whether HIF-1*α* is a regulatory signal of Rho GTPases and integrin *α*_4_, we analyzed the mRNA levels of HIF-1*α* and integrin *α*_4_ in BM-MSCs after the inhibition of HIF-1*α* by YC-1 treatment. The levels of HIF-1*α* mRNA in BM-MSCs were effectively decreased by YC-1 pretreatment under both normoxia and hypoxia (Figure 3(a)). However, the levels of mRNA transcription and protein expression of integrin *α*_4_ in BM-MSCs were increased when the cells were pretreated with YC-1 compared with controls regardless of oxygen concentration (Figures 3(b) and 3(c)). To verify the effect of HIF-1*α* on Rho GTPases, we analyzed the expression of ROCK1 and Rac1/2/3 in BM-MSCs after YC-1 pretreatment. The augmented expression of ROCK1 and Rac1/2/3 in BM-MSCs under hypoxia significantly declined with YC-1 pretreatment (Figure 3(d)). Therefore, these findings indicate that HIF-1*α* reduces the transcription and expression of integrin *α*_4_ and enhances the expression of ROCK1 and Rac1/2/3 in BM-MSCs under hypoxia.

### 3.4. Inhibition of Integrin *α*_4_ Promotes the Migration of BM-MSCs through Upregulation of MMP-2

**Figure 4:**
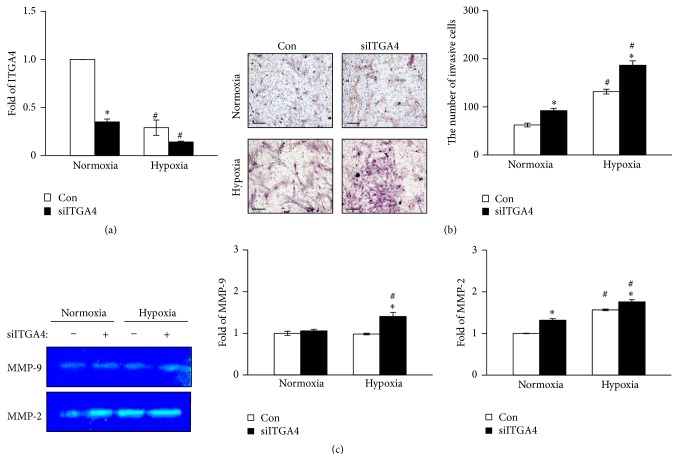
The effect of integrin *α*_4_ inhibition on BM-MSC migration and activities of MMPs under hypoxia. (a) The mRNA expression of integrin *α*_4_ in BM-MSCs was suppressed by transfection of integrin *α*_4_ siRNA. 18S rRNA was used as the loading control. (b) BM-MSC migration was significantly increased after siITGA4 transfection. Invasiveness of BM-MSCs was assessed by invasion assay (left). BM-MSCs invaded through the inserts were counted for quantification (right). (c) Enzymatic activities of MMP-9 and MMP-2 in BM-MSCs after siITGA4 transfection were determined by zymography (left). Quantification of enzymatic activities of MMP-9 (middle) and MMP-2 (right). ^*∗*^ *P* < 0.05 (compared with siITGA4 nontransfected group) and ^#^ *P* < 0.05 (compared with normoxic group). MMP: matrix metalloproteinase; siITGA4: integrin *α*4 siRNA.

We hypothesized that the expression of integrin *α*_4_, which is decreased by HIF-1*α* under hypoxia, may contribute to the migration of BM-MSCs. To confirm the effect of integrin *α*_4_ on the migration of BM-MSCs under hypoxia, we conducted knockdown experiments using siITGA4 under normoxia and hypoxia. The level of mRNA integrin *α*_4_ was significantly decreased by the transfection of siITGA4 under both hypoxic and normoxic conditions (Figure 4(a)). After siITGA4 treatment, the migration of BM-MSCs was revealed to be significantly increased under both normoxia and hypoxia (*P* < 0.05; Figure 4(b)). Moreover, enhanced migration induced by siITGA4 treatment was more evident under hypoxia compared with normoxia (*P* < 0.05; Figure 4(b)). Furthermore, the MMP-2 activity in BM-MSCs, which was elevated by hypoxia, significantly increased when the cells were transfected with siITGA4 (Figure 4(c)). While the MMP-9 activity was not altered by hypoxia, downregulation of integrin *α*_4_ by siITGA4 induced activation of MMP-9 under hypoxic conditions (Figure 4(c)). Taken together, downregulated integrin *α*_4_ enhances the migration of BM-MSCs through the stimulation of MMP-2 activity under normoxia as well as hypoxia.

### 3.5. Interaction between Integrin *α*_4_ and Hypoxia and Its Effect on the Rho GTPase Family

**Figure 5:**
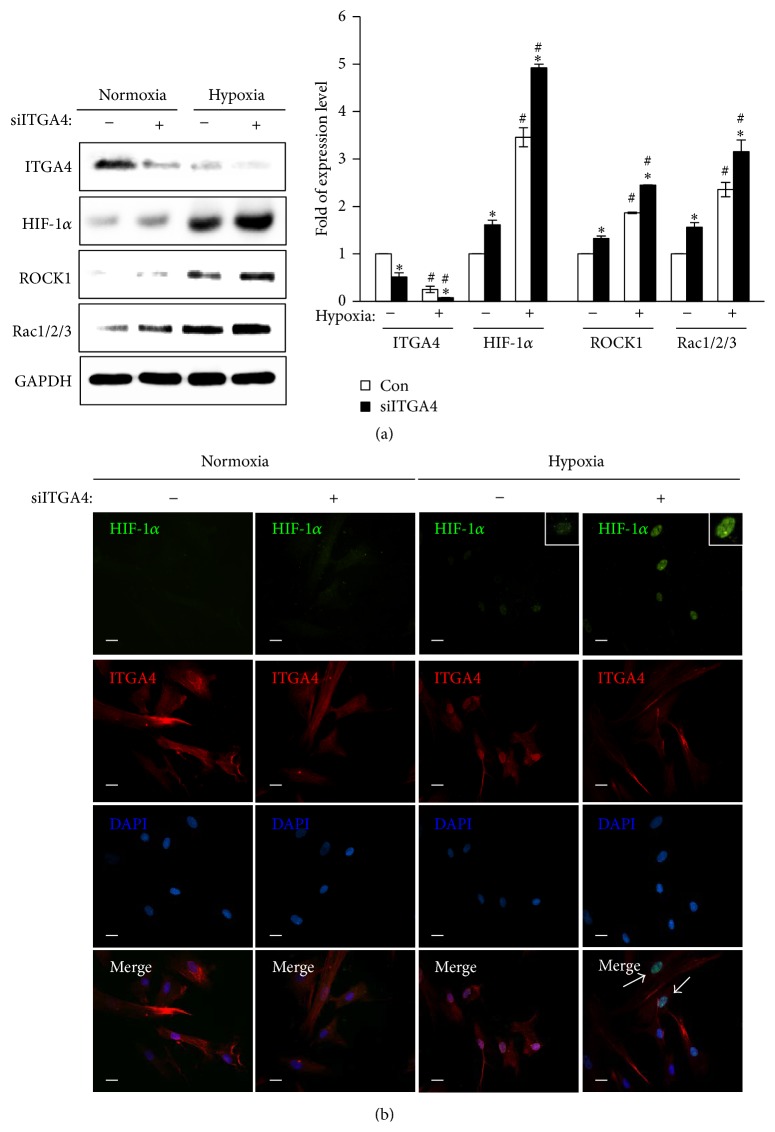
Interaction between integrin *α*_4_ and HIF-1*α* and its effect on expression of Rho GTPases under hypoxia. (a) Protein expression levels of integrin *α*_4_, HIF-1*α*, ROCK1, and Rac1/2/3 were assessed by Western blotting. GAPDH was used as the loading control. (b) HIF-1*α* and integrin *α*_4_ were localized with immunofluorescence in BM-MSCs after siITGA4 transfection under normoxic or hypoxic conditions. Blue: DAPI; green: HIF-1*α*; red: integrin *α*_4_. Scale bar = 80 *μ*m (400x original magnification). ^*∗*^ *P* < 0.05 (compared with siITGA4 nontransfected group) and ^#^ *P* < 0.05 (compared with normoxic group). DAPI: 4′,6-diamidino-2-phenylindole; HIF-1*α*: hypoxia-inducible factor-1*α*; ITGA4: integrin *α*_4_; ROCK1: Rho-associated kinase 1; siITGA4: integrin *α*_4_ siRNA.

To explore the possible interaction between integrin *α*_4_ and HIF-1*α* under hypoxia and its effect on the migration of BM-MSCs via Rho GTPases, we analyzed the expression levels of ROCK1, Rac1/2/3, and HIF-1*α* after siITGA4 transfection under normoxia and hypoxia. The expression of HIF-1*α*, ROCK1, and Rac1/2/3 was significantly increased by downregulated integrin *α*_4_ expression under both normoxia and hypoxia. Specifically, augmentation of their expression by siITGA4 was marked under hypoxic conditions compared with normoxic conditions (Figure 5(a)). HIF-1*α* was revealed to regulate the expression of integrin *α*_4_ and to be modulated by integrin *α*_4_, indicating the possible positive feedback between HIF-1*α* and integrin *α*_4_. Therefore, we assessed the translocation of HIF-1*α* in BM-MSCs under hypoxia using immunofluorescence. HIF-1*α* translocated into the nucleus of the cells under hypoxia, whereas it was located in cytoplasm under normoxia. Furthermore, the translocation of HIF-1*α* into the nucleus became obvious with the knockdown of integrin *α*_4_ by siRNA transfection (Figure 5(b)). These results suggest that HIF-1*α* and integrin *α*_4_ interact and regulate each other and modulate the dynamic expression of the Rho GTPase family leading to the regulation of BM-MSC migration.

## 4. Discussion

Stem cell therapy using MSCs has been explored for the treatment of various degenerative diseases. However, the mechanisms of action are poorly understood. MSC migration is a critical factor determining the efficacy of stem cell therapy because the therapeutic effect of MSCs can only be expected after the proper engraftment of transplanted MSCs to the damaged tissues. Ceradini and colleagues reported that the recruitment of progenitor cells into the regenerating tissues was regulated by hypoxic gradients via the HIF-1 induction of SDF-1, which binds to CXCR4 on circulating progenitor cells [27]. In a previous clinical study, it was demonstrated that MSC coinfusion improved hematopoietic stem cell engraftment through restoration of a normal level of SDF-1 in 8 patients with acute myeloid leukemia undergoing hematopoietic stem cell transplantation [28]. However, the interactions between HIF-1*α* and the signaling molecules, such as integrins, MMPs, and Rho GTPases, under hypoxia and their influences on MSC migration have not been fully elucidated.

In the present study, we verified that hypoxia-activated HIF-1*α* enhanced the migration of BM-MSCs. Furthermore, the hypoxia-induced enhancement of BM-MSC migration was diminished after inhibition of HIF-1*α*. These results suggest that HIF-1*α* may be a crucial regulatory signal in facilitating BM-MSC migration under hypoxic conditions. If HIF-1*α* is activated under hypoxic conditions, HIF-1*α* translocates into the nucleus and activates the transcription of genes involved in cell survival, differentiation, and migration [29]. In the present study, the translocation of HIF-1*α* into the nucleus of BM-MSCs was demonstrated under hypoxia and additionally became marked after the knockdown of integrin *α*_4_. We also identified that the expression of integrin *α*_4_ was decreased by HIF-1*α* under hypoxic conditions. Therefore, the results of our study implicate the possible existence of a positive feedback loop between HIF-1*α* and integrin *α*_4_. Integrins are transmembrane proteins known to regulate a variety of physiologic events, such as cell growth, differentiation, and migration [30, 31]. In a previous study, the expression of integrin *α*_5_ was determined to increase under hypoxic conditions, resulting in the enhancement of extravillous trophoblast cell migration during early pregnancy [32]. Moreover, it was demonstrated that HIF-1*α*-induced upregulation of integrin *α*_5_ mediated cancer cell invasion [33]. Although we previously reported the effect of HIF-1*α*-induced integrin *α*_4_ suppression on trophoblast invasion [34], HIF-1*α*-induced downregulation of integrin *α*_4_ and the positive feedback loop between HIF-1*α* and integrin *α*_4_ in BM-MSCs exposed to hypoxia have not been reported so far.

MMPs are proteases that degrade the ECM proteins and play a major role in cancer invasion and metastasis [35]. It was reported that hypoxia-activated HIF-1*α* reduced E-cadherin expression and augmented MMP-2 expression during cancer cell migration [36]. Therefore, according to the results of the present study, BM-MSC behavior may resemble those of cancer cells with respect to augmenting cellular invasiveness via MMP-2 activation under hypoxia. The activities of MMP-9 as well as MMP-2 in trophoblast cells increased under hypoxic conditions in the aforementioned study [34]. In our study, MMP-2 activity was significantly enhanced under hypoxia contributing to increased BM-MSC migration as a result of suppressing integrin *α*_4_, whereas MMP-9 activity was not altered. This finding suggests that enhancement of BM-MSC migration under hypoxia is attributed to augmented intratissue migration via MMP activity, especially MMP-2, as well as cell adhesion via adhesion molecules.

FAK, a cellular focal adhesion-associated protein kinase, is known to be involved in cell adhesion and migration [37, 38]. Skuli et al. showed that HIF and reduced oxygen tension increased the expression of integrin *α*_5_ *β*_3_ leading to increased migration of trophoblast stem cells through FAK activation [39]. In addition, integrin *α*_5_ *β*_1_ was identified to mediate MSC migration during vascular remodeling by inducing FAK activity and platelet-derived growth factor receptor-*β* phosphorylation [40]. Tyrosine phosphorylation of FAK in response to growth factor stimulation and integrin engagement was shown to trigger phosphorylation of paxillin, which regulates Rho GTPases [41]. Based on this evidence, we hypothesized that FAK and Rho GTPases may be negatively regulated by integrin *α*_4_. The knockdown of integrin *α*_4_ by siRNA transfection induced upregulation of Rac1/2/3 and ROCK1 and changes in their expression levels were marked under hypoxic conditions. However, FAK and RhoA, which are upstream signals of ROCK1, were not significantly changed by inhibition of integrin *α*_4_ (data not shown). Therefore, these findings indicate that increased expression of Rac1/2/3 and ROCK1 as a result of integrin *α*_4_ suppression under hypoxia stimulates the migration of BM-MSCs, whereas tyrosine phosphorylation of FAK was not induced.

## 5. Conclusions

In summary, our study demonstrated that HIF-1*α*-mediated downregulation of integrin *α*_4_ facilitated the migration of BM-MSCs under hypoxia via MMP-2 activation and enhanced expression of Rac1/2/3, which belongs to the Rho GTPase family, and ROCK1, which is a downstream signal of integrins and Rho GTPases. Moreover, the possible existence of a positive feedback loop between HIF-1*α* and integrin *α*_4_ was revealed. Although further* in vivo* studies are needed to confirm the influences of the interactions between HIF-1*α*, integrin *α*_4_, and the Rho family of GTPases on BM-MSC homing and migration, stabilization of HIF-1*α* or knockdown of integrin *α*_4_ in BM-MSCs by genetic manipulation may be a potential therapeutic approach to enhancing the efficacy of stem cell therapy using BM-MSCs.

## Abbreviations

MSC: Mesenchymal stem cells
BM-MSCs: Bone marrow-derived mesenchymal stem cells
SDF-1: Stromal cell-derived factor 1
ECM: Extracellular matrix
HIF-1*α*: Hypoxia-inducible factor-1*α*
CXCR4: C-X-C chemokine receptor type 4
ROCK: Rho-associated kinase
FBS: Fetal bovine serum
siRNA: Short interfering RNA
siITGA4: Integrin *α*_4_ siRNA
PCR: Polymerase chain reaction
PBS: Phosphate-buffered saline
DAPI: 4′,6-Diamidino-2-phenylindole
SDS-PAGE: Sodium dodecyl sulfate polyacrylamide gel electrophoresis
FAK: Focal adhesion kinase
MMP: Matrix metalloproteinase.
